# Coronary Artery Spasm: Risk Factors, Pathophysiological Mechanisms and Novel Diagnostic Approaches

**DOI:** 10.31083/j.rcm2305175

**Published:** 2022-05-16

**Authors:** Zijie Lin, Xinyi Lin, Xin Zhao, Chenchao Xu, Bokang Yu, Yiwen Shen, Liliang Li

**Affiliations:** ^1^Department of Forensic Medicine, School of Basic Medical Sciences, Fudan University, 200032 Shanghai, China; ^2^Department of Cardiology, Shanghai Institute of Cardiovascular Diseases, Zhongshan Hospital, Fudan University, 200032 Shanghai, China

**Keywords:** coronary artery spasm, risk factors, endothelial dysfunction, vascular smooth muscle cell hyperreactivity, adventitial inflammation, diagnostic approaches

## Abstract

Coronary artery spasm (CAS) is a transient reversible subtotal or complete occlusion induced by coronary hypercontraction and the critical cause of myocardial ischaemia with non-obstructive coronary arteries. During the past decades, our knowledge of the risk factors and pathophysiological mechanisms of CAS have been increasingly progressed, and various diagnostic approaches, including imaging technologies and novel biomarkers, have been proposed to serve well to diagnose CAS clinically. This review aims to summarize these research progresses on the risk factors of CAS and introduce current knowledge about the mechanisms accounting for CAS, including endothelial dysfunction, vascular smooth muscle cell hyperreactivity, and adventitial and perivascular adipose tissue inflammation. We also gathered the recently evolved diagnostic approaches and analyzed their advantages/disadvantages, in purpose of enhancing the diagnostic yield on the basis of ensuring accuracy.

## 1. Introduction

In 1959, Prinzmetal *et al*. [1] first proposed the term “variant angina” which is later evolved and re-named as coronary artery spasm (CAS). CAS is generally considered as abnormal contraction of epicardial coronary arteries causing myocardial ischemia and includes microvascular CAS in a broad sense. Clinically, CAS is defined as a transient reversible subtotal or complete occlusion of coronary arteries with >90% vasoconstriction on angiography using spasm provocation test (SPT) known as the gold standard approach, accompanied by angina pectoris and ischaemic electrocardiogram (ECG) changes [2]. CAS could also appear in common ischemic heart disease, including stable angina, unstable angina, and acute myocardial infarction (AMI), coupled with a variety of pathophysiological alterations, such as coronary atherosclerosis and thrombosis. The current European Society of Cardiology (ESC) guideline further emphasizes the concept that vasospastic angina (VSA) and microvascular angina are also components of chronic coronary syndrome (CCS) [3]. Moreover, coronary angiography (CAG) revealed that the degree of stenosis due to mere atheromatosis was less than 50% in a large angina patient cohort [4], suggesting the additional involvement of CAS in coronary stenosis and the importance of assessing CAS in patients with CCS.

CAS is not a benign disease. Approximately 1–14% of AMIs are considered to occur in CAS patients, which could further lead to fatal arrhythmia, and even sudden cardiac death [5]. Thrombosis secondary to CAS may be another important cause of myocardial infarction [6]. Despite the area of CAS-induced myocardial infarction is small in general, spontaneous reperfusion after CAS subsiding also increases the risk of fatal arrhythmia [7]. VSA is the major clinical manifestation of CAS-induced myocardial ischemia. It is usually independent of effort occurring at rest with obvious circadian rhythm, namely more occurrences in the period from midnight to dawn [2]. ST-segment elevation or depression on ECG is one of the clinical features [2]. Compared with coronary atherosclerotic diseases (CAD), CAS is more prevalent in women, younger people and Asian populations, such as Japanese and South Koreans. With the utilization of invasive SPT, it is also not uncommon for VSA in some Western countries such as Germany and Australia [8, 9]. However, true prevalence needs further investigation due to the rare utilization of SPT in most countries, such as China, where SPT is cautiously performed only for clinical diagnosis in specialized medical centers.

Recent years have witnessed increasing advances towards our understanding of CAS. This review aims to introduce the recent knowledge on the risk factor, pathophysiological mechanisms of CAS and also highlights the latest advancements in clinical diagnosis of CAS, aiming at providing effective alternatives for invasive methods, especially for the countries where SPT is not performed routinely in the clinic.

## 2. Precipitating Factors and Clinical Risk Factors 

**Fig. 1.**
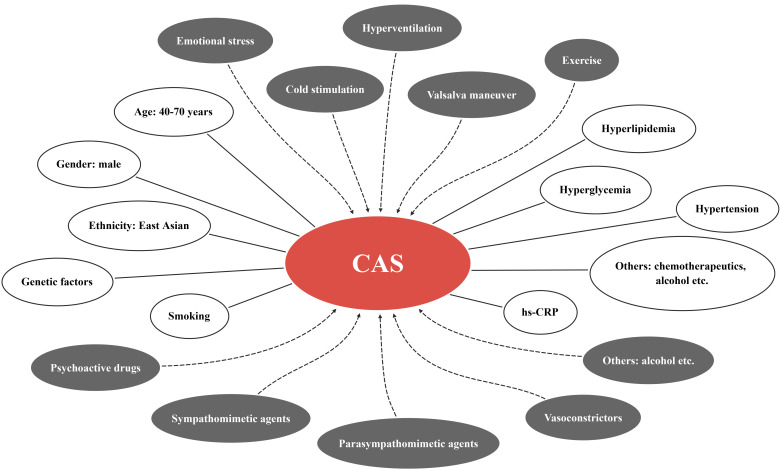
**Precipitating factors (grey ellipses) and clinical risk factors (white ellipses) of CAS**. CAS, coronary artery spasm; hs-CRP, high-sensitivity C-reactive protein.

There are a vast number of precipitating factors for CAS (Fig. 1), which can be divided as physiological and pharmacological categories. The former includes emotional stress, cold stimulation, hyperventilation, valsalva maneuver, and exercise etc., while the latter contains psychoactive drugs (such as cocaine, marijuana, and amphetamine), sympathomimetic agents (such as epinephrine, norepinephrine), parasympathomimetic agents (such as acetylcholine (Ach), pilocarpine), vasoconstrictors (such as thromboxane, ergonovine), alcohol consumption, and magnesium deficiency etc. [10, 11]. In addition, there have been reports about CAS induced by traditional Chinese medicine, including Di-Long (dried earthworm), Ma-Huang (plant of ephedra), and cucumis polypeptide (the combined extracts from deer horn and sweet melon seeds) [12].

Unlike CAD, CAS patients seem to be more common among young people and women [7, 13]. However, male patients still account for the majority of CAS patients, and high prevalence is in the age range of 40–70 years [4]. As mentioned above, CAS is a highly prevalent disease in East Asia with ethnic and genetic diversity. It is worth noting that East Asian patients tend to present diffuse and multi-vascular CAS, while Caucasians tend to present focal CAS [14]. Smoking is an unequivocal risk factor for CAS and about 75% of CAS patients are smokers [15]. It was also reported that the proportion of smokers in CAS patients was 42.6%, but it still surpassed that in CAD patients [16]. The substances in cigarettes, such as carbon monoxide and nicotine, are able to damage blood vessels by increasing inflammation and oxidative stress, which explains why smoking is a high risk factor for CAS [17]. Although hyperlipidemia, hyperglycemia, and hypertension in CAS patients are less common than those in CAD patients [16], these metabolic disorders also contribute to the development of CAS. Serum high-sensitivity C-reactive protein (hs-CRP) is higher in CAS patients than that among healthy individuals, implicating the potential of hs-CRP to be a predictor of CAS [18]. Moreover, alcohol consumption [19] and chemotherapeutics [20] that destruct blood vessels through independent mechanisms have also been found to relate to CAS.

## 3. Pathophysiological Mechanisms of CAS

**Fig. 2.**
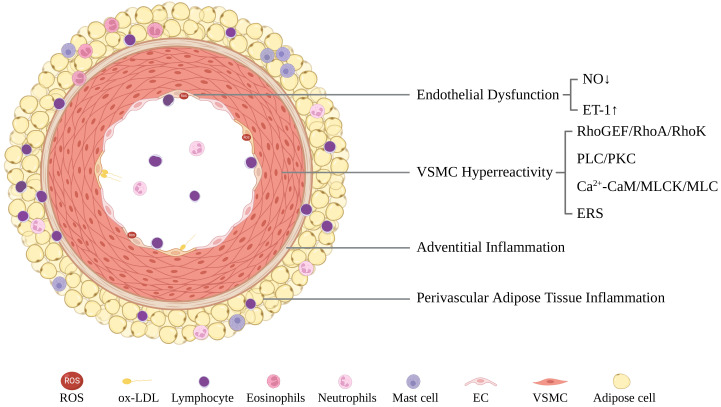
**A schematic illustration of CAS pathogenesis including endothelial dysfunction, VSMC hyperreactivity, and adventitial/perivascular adipose tissue inflammation**. CaM, calmodulin; EC, endothelial cell; ERS, endoplasmic reticulum stress; ET-1, endothelin-1; MLC, myosin light chain; MLCK, MLC kinase; NO, nitric oxide; ox-LDL, oxidized low-density lipoprotein; PKC, protein kinase C; PLC, phospholipase C; RhoA, Ras homolog gene member A; RhoGEF, Rho guanine nucleotide exchange factors; RhoK, Rho kinase; ROS, reactive oxygen species; VSMC, vascular smooth muscle cell.

The pathogenesis of CAS is complicated and could be categorized as endothelial dysfunction (ED) in the intima, vascular smooth muscle cell (VSMC) hyperreactivity in the media, and adventitial and perivascular adipose tissue (PVAT) inflammation (Fig. 2).

### 3.1 Endothelial Dysfunction in the Intima

ED is defined as a series of phenotypes related to pathophysiological heterogeneous changes in vascular tone, permeability, inflammation, and de-differentiation by the ESC [21]. Clinical observations have shown that ED is associated with the pathogenesis of CAS. Nitroglycerin and isosorbide dinitrate, two endothelial-independent vasodilators, are highly efficient to relieve vasospasm angina during CAS [22, 23]. Nitrates are even prescribed as vasodilator agents after SPT [24]. In clinical angiography, it has been found that most of the spastic sites were in parallel to atherosclerotic plaque [25], and the coronary intima of CAS patients was remarkably thickened [26]. Immunohistological analysis of endomyocardial biopsy samples further showed that most CAS patients had endothelial cells (ECs) activation [27]. After removal of the endothelium, porcine coronary arteries successfully developed CAS with high cholesterol feeding [28].

At the molecular level, ED refers to disruption of homeostasis for endothelial regulation of vascular tension, and defines the abnormal function of synthesis and release of vasoactive substances such as nitric oxide (NO) and endothelin-1 (ET-1). Endothelial NO synthase (eNOS) dimer is the pivotal molecule for ECs to physiologically produce NO. When high-risk factors are present, reactive oxygen species (ROS) is increased in ECs due to stimulation by reduced nicotinamide adenine dinucleotide phosphate (NADPH) oxidase [21, 29]. The increased ROS then clears NO in ECs and converts it into peroxynitrite (${\mathrm{ONOO}}^{-}$) with strong oxidizing property. Increased ROS also oxidizes tetrahydrobiopterin (${\mathrm{BH}}_{4}$), an important co-factor of eNOS that maintains its dimerization, to be dihydrobiopterin (${\mathrm{BH}}_{2}$), leading to eNOS uncoupling and attenuation of NO synthesis. Furthermore, production of the eNOS monomer can in turn prevent $O_{2}$ from converting into superoxide anion ($O^{2-}$), which further augments ROS and exacerbates the failure of eNOS dimerization. In addition, increased ROS also mediates the development of inflammation and ECs damage, which ultimately leads to ED. ET-1 is a powerful vasoconstrictor, and the increase of its synthesis and release is also one of the components of ED. Toyo-oka *et al*. [30] presented that plasma ET-1 level of CAS patients was significantly higher than that of non-CAS patients. Increased ET-1 then activated protein kinase C (PKC) and thereby enhancing coronary contraction induced by prostaglandin F2α (PGF2α) and 5-hydroxytryptamine (5-HT; also named as serotonin) [31, 32]. High levels of ET-1 also repressed the NO synthesis in a PKC-dependent manner [33]. Studies have shown that cigarette smoking increased the vascular ET-1 receptors by activating mitogen-activated protein kinase (MAPK) [34]. In addition, cocaine may promote the release of ET-1 to elicit CAS [35], and a few hours after drinking, CAS was observed to be caused by an obvious elevation of ET-1 level [36]. These studies might explain the association between common risk factors and the development of ED-related CAS.

The ED in association with CAS is further supported by genetic evidence. The polymorphisms of *NOS* gene [37], aldehyde dehydrogenase 2 (*ALDH2*) gene [38], *paraoxonase I* gene [39], *p22 phox* gene in male [40], manganese superoxide dismutase (*MnSOD*) gene [41], and inflammatory factor interleukin-6 (*IL-6*) gene [40] all influence the NO synthesis, oxidative stress and inflammation. The polymorphisms of *ET-1* gene are also related to CAS. Lee *et al*. [42] showed that CAS is related to the + 138delA, G8002A and Lys198Asn polymorphisms of the *ET-1 *gene. Ford *et al*. [43] observed that patients with coronary microvascular dysfunction have a higher frequency of the rs9349379-G allele and are associated with higher serum ET-1 levels.

Of note, Shimokawa [44] and Lanza *et al*. [45] presented evidence such as successful establishment of CAS animal models with normal endothelial function to show that ED might not be the key mechanism of CAS pathogenesis. Moreover, some CAS patients were resistant to nitrate treatment, which means supplementing NO cannot always mitigate CAS [46]. In addition, not all CAS patients have ED, and ED or inhibition of NO synthesis alone may be insufficient to cause CAS [45, 47], implicating that ED is an important yet unnecessary pathophysiological change of CAS.

### 3.2 VSMC Hyperreactivity in the Media

While VSMC hyperreactivity is dependent on the cytoplasm ${\mathrm{Ca}}^{2+}$ sensitivity or the [${\mathrm{Ca}}^{2+}$]_i_ quantity, multiple pathways such as RhoGEF/RhoA/RhoK pathway, PLC/PKC pathway, ${\mathrm{Ca}}^{2+}$-CaM/MLCK/MLC pathway, and endoplasmic reticulum stress have been suggested to regulate the VSMC hyperreactivity and induce CAS.

#### 3.2.1 RhoGEF/RhoA/RhoK Pathway

The Ras homolog family (Rho) pathway activity has been observed to have circadian rhythm, showing higher activity particularly at midnight and early morning [48, 49], a time window that conforms to the circadian rhythm of CAS. Also in CAS patients, intervention by Rho kinase (RhoK) inhibitors remarkably reduced Ach-induced coronary contraction [50, 51], as well as the degree of myocardial ischemia [52, 53], and further improve coronary artery relaxation combined with nitroglycerin [54]. These data suggested that the Rho pathway plays a pivotal role in the pathogenesis of CAS in human. Indeed, Rho guanine nucleotide exchange factors (RhoGEFs) are a class of molecules with abundant subtypes, which can activate Rho protein by converting GDP into GTP [55]. In VSMCs, RhoGEFs are mainly regulated by G protein-coupled receptors (GPCRs) and the activated RhoGEFs then transduce signals to the downstream Rho family member A (RhoA), thereby modulating the ${\mathrm{Ca}}^{2+}$ sensitivity [55, 56, 57].

Many etiologies can induce VSMC hyperreactivity by activating the RhoA/RhoK pathway, such as oxidized low-density lipoprotein (oxLDL) [58, 59], chronic hypoxia and ROS [60, 61, 62], inflammation [63, 64], hemorrhagic shock [65], and chronic stress [66]. Galle *et al*. [58] observed that oxLDL augmented the activity of RhoA in rabbit aorta, and thereby potentiating the contractile responsiveness of aorta to Angiotensin (Ang) II. Bolz *et al*. [59] proved that oxLDL increased the [${\mathrm{Ca}}^{2+}$]_i_ and RhoK-mediated ${\mathrm{Ca}}^{2+}$ sensitization in isolated small resistance arteries, which reduced the response to vasodilators and provoked vascular hyperreactivity to norepinephrine and Ach. Maruko *et al*. [60] showed that chronic hypoxia attenuated [${\mathrm{Ca}}^{2+}$]_i_ in coronary artery of fetal sheep, but enhanced ${\mathrm{Ca}}^{2+}$ sensitivity, and thromboxane A2 (${\mathrm{TXA}}_{2}$) receptor-mediated contraction could be inhibited by Rho inhibitors rather than PKC inhibitors. Gao *et al*. [61] further showed that hypoxic stimulus increased the levels of intracellular inosine 5’-triphosphate (ITP) and inosine 3’,5’-cyclic monophosphate (cIMP), which promoted the elevation of RhoK activity. Knock *et al*. [62] showed that ROS mediated ${\mathrm{Ca}}^{2+}$ sensitization through the RhoK pathway in VSMCs. Inflammatory factors could also enhance the expression and activation of RhoK as well as its downstream molecules in human coronary VSMCs [64]. Corticosteroids play significant roles in the treatment of refractory CAS patients, and researchers believed that it might be attributed to the inhibition of inflammation and alleviation of the coronary VSMC hyperreactivity [67, 68]. In COVID-19 patients with cytokine storms, several cases of severe CAS have also been reported [69, 70], but it is unknown whether these patients suffered from CAS before infection of SARS-CoV-2. It should be noted that chronic inflammation and oxidative stress are extremely common in cardiovascular diseases, especially CAD, but not all patients will develop CAS. We believe that these factors are in association with but rather independent causes of VSMC hyperreactivity.

Polymorphisms of* RhoK* gene also link with CAS. Kamiunten *et al*. [71] found that the missense mutation G930T resulted in the enhancement of RhoK activity in CAS patients and Yoo *et al*. [72] found that the GTCTG haplotype in 5 interesting single nucleotide polymorphisms (SNPs) might play a protective role in non-CAS patients.

Myosin light chain (MLC) phosphatase (MLCP) is one of the most important downstream molecules of RhoK and its inactivation by RhoK enhances the phosphorylation of MLC. Phosphorylated MLC (pMLC) was found at the spastic sites and positively correlated with the degree of contraction in interleukin 1β (IL-1β)-induced porcine CAS model [73], further supporting the involvement of Rho pathway in the development of CAS.

#### 3.2.2 PLC/PKC Pathway

Okumura *et al*. [74] cultivated the skin fibroblasts from CAS patients and found that the phospholipase C (PLC) activity was enhanced and positively correlated with the contractile hyperresponsiveness of coronary arteries, proposing that the increased PLC activity may be involved in the pathogenesis of CAS. The p122 protein, an agonist of PLC, was up-regulated in skin fibroblasts of CAS patients [75]. Increased p122 protein promoted the basal and peak [${\mathrm{Ca}}^{2+}$]_i_ to Ach in human coronary VSMCs [75]. Also, in* p122* transgenic mice, ergonovine could successfully induce the occurrence of CAS [76]. Nakano *et al*. [77] further found that the R257H mutation in the *PLC-δ1* gene was higher in CAS patients, though the incidence was overall less than 10%. In the R257H homozygous knock-in mice, 3 in 5 (60%) developed CAS using the microvascular filling technology [78].

In addition, downstream PKC is also critically involved in the development of CAS [79, 80]. Giardina *et al*. [81] proved that oxLDL enhanced the ${\mathrm{Ca}}^{2+}$ sensitivity of VSMCs by activating PKC-α and PKC-ϵ. Allahdadi *et al*. [82] treated rats with eucapnic intermittent hypoxia, leading to contractile hyperresponsiveness to ET-1 *via* PKCδ in the small mesenteric arteries. In support of this, the downstream signals of PKC such as C-kinase potentiated protein phosphatase-1 inhibitor of 17 kDa (CPI-17), calponin (CaP), MAPKs were also revealed to regulate VSMC hyperreactivity. CPI-17, upon phosphorylation by PKC, inhibits the activity of the catalytic subunit PP1cδ of MLCP, leading to inactivation of MLCP. In *CPI-17* knockout mice, the systolic blood pressure and average blood pressure decreased apparently, and vascular contraction induced by various agonists was significantly weakened [83, 84]. These results indicate that CPI-17 might be one of the most important downstream factors boosting vasoconstriction. A study also showed that inhibition of CaP binding to actin would augment ${\mathrm{Ca}}^{2+}$ sensitivity of vascular smooth muscle in isolated mesenteric artery [85]. However, this was challenged by follow-up studies that showed knockout of *CaP* gene did not affect the ${\mathrm{Ca}}^{2+}$ sensitivity in mice [86]. p38 MAPK might also be involved in PKC-regulated contractile responsiveness since adenosine increased pMLC level through the p38 MAPK/MK2 pathway, leading to enhancement of VSMC responsiveness to AngII [87].

Interestingly, in the porcine CAS model induced by IL-1β, RhoK inhibitor was capable of repressing the effect of PKC agonist, but the effect of RhoK could not be blocked by PKC inhibitors [88], implying that the RhoK may also be downstream of PKC signaling in the development of CAS.

#### 3.2.3 Calcium and ${\mathrm{Ca}}^{2+}$-CaM/MLCK/MLC Pathway

Calcium channel blockers (CCBs) have been well established as therapeutic agents for CAS in clinic, suggesting that ${\mathrm{Ca}}^{2+}$ is the core element of CAS. Indeed, the up-regulation of voltage-dependent ${\mathrm{Ca}}^{2+}$ channels and enhanced ${\mathrm{Ca}}^{2+}$ influx are major features of hypertension [89]. Smith *et al*. [90] observed that the V734I mutation of *ABCC9* gene (encoding Sur2 subunit of the $K_{\text{ATP}}$ channel) was associated with CAS. When Sur2 subunit of the $K_{\text{ATP}}$ channel was knocked out, the function of ${\mathrm{Ca}}^{2+}$ channels was perturbed, leading to spontaneous CAS episodes [91].

Calcium functions *via *binding with Calmodulin (CaM). The ${\mathrm{Ca}}^{2+}$-CaM complex then directly activates the MLC kinase (MLCK). Decreased MLCK activity attenuated ${\mathrm{Ca}}^{2+}$ sensitivity and contractile responsiveness in carotid arteries [92]. Kim [93] observed that CPI-17 and MLCK were up-regulated in obese Sprague-Dawley rats fed with high-fat, which collectively mediated the vascular hyperreactivity. ${\mathrm{Ca}}^{2+}$/CaM-dependent protein kinase II (CaMKII) activated by ${\mathrm{Ca}}^{2+}$-CaM also promotes the activation of MLCK through the extracellular signal-regulated kinase 1 and 2 (${\mathrm{ERK}}_{1/2}$) at a slow rate, but it phosphorylates a specific serine residue in the CaM-binding domain of MLCK, which reduces the ${\mathrm{Ca}}^{2+}$ sensitivity of MLC phosphorylation [94]. Moreover, autophosphorylation on CaMKII Thr286 greatly enhances its affinity with CaM, which is involved in maintaining vasoconstriction [95]. It is suggested that the abnormal activity of CaMKII may also be involved in the VSMC hyperreactivity. Furthermore, we also performed immunohistochemistry analysis of death cases from CAS and confirmed that pMLC2 might serve as a tissue marker of antemortem CAS [96].

#### 3.2.4 Endoplasmic Reticulum Stress

Endoplasmic reticulum stress (ERS) is defined as the accumulation of unfolded and/or misfolded proteins in the endoplasmic reticulum (ER) that breaks the ER homeostasis, and thereby activating the unfolded protein response (UPR) to restore and maintain the ER homeostasis [97]. The causes of ERS encompass various physiological or pathological stimuli such as hypoxia, starvation, oxidative stress, imbalance of ${\mathrm{Ca}}^{2+}$ homeostasis, etc. [97]. The UPR proceeds through three signaling pathways to resist the cellular stress, including transcription factor 6 (ATF6) pathway, inositol-requiring enzyme 1 (IRE1) pathway, and protein kinase R-like ER kinase (PERK) pathway [97].

Choi *et al*. [98] found that hyperglycemia led to enhanced coronary myogenic response and ED *via* triggering ERS in mice. Liang *et al*. [99] showed that ERS inducers, such as tunicamycin (Tm), increased the phosphorylation of MLC in VSMCs and enhanced the contractile responsiveness to phenylephrine in aorta independent of endothelium. Zhang *et al*. [100] observed that ceramide resulted in the VSMC hyperreactivity to phenylephrine through ERS/COX-2/PGE2 pathway. We observed that an ERS inhibitor significantly prevented VSMC contraction, whereas Tm aggravated the CAS-induced myocardial ischemia in mice, and ERS regulated CAS possibly through the MLCK/MLC pathway [101]. Ziomek *et al*. [102] also pointed out that Tm did not activate ${\mathrm{Ca}}^{2+}$ channels, but altered the ${\mathrm{Ca}}^{2+}$ permeability of plasma membrane and ER, leading to an increase in [${\mathrm{Ca}}^{2+}$]_i_ and initiating the VSMC contraction. Meanwhile, Tm also caused a decrease of ${\mathrm{Ca}}^{2+}$ concentration in ER [99, 102]. The above studies have shed novel insights into the pathogenesis of CAS. However, the detailed mechanisms of how ERS regulated CAS remain largely unknown and merit future investigation.

### 3.3 Adventitial and PVAT Inflammation 

Shimokawa and colleagues utilized IL-1β and other inflammatory factors to mediate coronary adventitial inflammation and established a porcine CAS model [63, 103], indicating that adventitial inflammation is able to induce CAS. Coronary adventitial infiltration of mast cells and/or eosinophils in some CAS autopsy reports also suggested the influence of adventitial inflammation on the pathogenesis of CAS [104, 105], but mast cells are likely to provoke CAS by releasing histamine and other vasoconstrictors [106]. In recent years, PVAT inflammation in the pathogenesis of CAS has been brought to the forefront of research interest. Ohyama *et al*. [107] observed an increased coronary PVAT volume of CAS patients using CTA technique, which was in general consistent with Ito *et al*. [108]. The increased PVAT inflammation was further evidenced by remarkable ^18^F-fluorodeoxyglucose (FDG) uptake *via* positron emission tomography/computed tomography (PET/CT) scanning in CAS patients [109]. Nishimiya *et al*. [110] also noticed an enhanced formation of adventitial vasa vasorum in CAS patients using optical frequency domain imaging, and the extent of adventitial vasa vasorum positively correlated with RhoK activity of circulatory leukocytes. Moreover, drug-eluting stent-induced CAS was also observed at the presence of PVAT inflammation in a porcine model [111].

Of note, the vasoconstriction effect of PVAT inflammation seems to be VSMCs-dependent [112]. For instance, Lynch *et al*. [113] revealed that PVAT activated the ${\mathrm{BK}}_{C\,a}$ channels on VSMCs by releasing adiponectin, thereby resisting vasoconstriction. Saxton *et al*. [114] found that sympathetic excitation triggered the release of adiponectin from PVAT *via*β_3_-adrenergic receptors, and PVAT took up norepinephrine, which prevented its interaction with VSMCs. Aalbaek *et al*. [115] proved that PVAT inhibited the ${\mathrm{Ca}}^{2+}$ sensitivity mediated by the RhoK pathway in the coronary artery of rats, further validating that PVAT is capable of regulating the ${\mathrm{Ca}}^{2+}$ sensitivity of coronary VSMCs.

## 4. Novel Diagnostic Approaches

In the clinic, CAS may present in a variety of ways and is often asymptomatic, which causes CAS remaining a quite underdiagnosed and underreported disease with an average estimated delay of 3 months from presentation to diagnosis [7]. Currently, it is an urgency to develop accessible and practical diagnosis approaches for the disease. This section will introduce state-of-the-art diagnostic approaches (Tables 1,2) that might aid in clinical diagnosis of CAS.

**Table 1.** **A summary of the imaging approaches for diagnosis of CAS**.

Imaging approaches	Advantages	Disadvantages	References
Coronary angiography (CAG)	Gold standard when performed under provocation testing	Confusion between CAD and CAS	[2, 116, 117, 118]
		Omission in conditions of severe stenosis	
Electrocardiogram (ECG)	Convenience, safety, availability, acceptability	Low specificity	[119, 120, 121, 122, 123]
		Omission in resting intervals	
Intracoronary imaging approaches	Exhibition of morphological and functional changes despite complex conditions	In theoretical stage	[117, 119, 124, 125, 126, 127, 128]
		High requirements for equipment and operators	
*OCT*	Better image quality and resolution to estimate intima	Interruption of the blood flow	[126, 128]
		Tissue penetration: 2 mm	
		Safety worries	
*IVUS*	Deeper penetration (4–8 mm) for accessing perivascular injury without interrupting the blood flow	Less resolution	[126, 129]
Positron emission tomography (PET)	Revelation of coronary vasomotor function and tissue image	Expensive	[109, 130]
		High requirements for equipment	
*${\mathrm{18}}^{F}$-PET*	Evaluation of inflammation of coronary perivascular adipose tissue	Expensive	[109]
		High requirements for equipment	
Myocardial contrast echocardiography (MCE)	Microvascular evaluation	Indirect functional information	[131, 132, 133]
		Ignorance of minor systolic wall move	
		Low resolution	

OCT, optical coherence tomography; IVUS, intravascular ultrasound.

**Table 2.** **A summary of the novel diagnostic biomarkers in CAS**.

Markers	Category	References
cystatin C	Endothelial dysfunction	[141, 142, 143, 144, 145]
xanthine oxidoreductase (XOR)	Endothelial dysfunction	[29, 146, 147, 148]
hs-CRP	Inflammation	[18, 148, 149, 150]
sCD40L	Inflammation	[18]
peripheral monocyte counts	Inflammation	[151]
Endothelin-1 (ET-1)	Vasomotor	[30, 152]
Serotonin (5-HT)	Vasomotor	[153, 154]
Neuropeptide Y	Vasomotor	[141, 155]
Lipoprotein(a)	perivascular adipose tissue metabolism	[148, 156, 157, 158, 159]
RhoK activity in circulating neutrophils	RhoK pathway	[49, 50, 66, 160, 161, 162, 163, 164, 165]
pMLC2	Vascular smooth muscle cell hypersensitivity	[96, 101]
ox-LDL	Oxidative stress	[166, 167]
MDA-LDL	Oxidative stress	[166, 168, 169]
miR-17-5p, miR-92a-3p, miR-126-3	MicroRNAs	[170, 171, 172, 173]

### 4.1 Imaging Approaches (Table 1, Ref. [2, 109, 116, 117, 118, 119, 120, 121, 122, 123, 124, 125, 126, 127, 128, 129, 130, 131, 132, 133])

#### 4.1.1 Spasm Provocation Test (SPT)

**Fig. 3.**
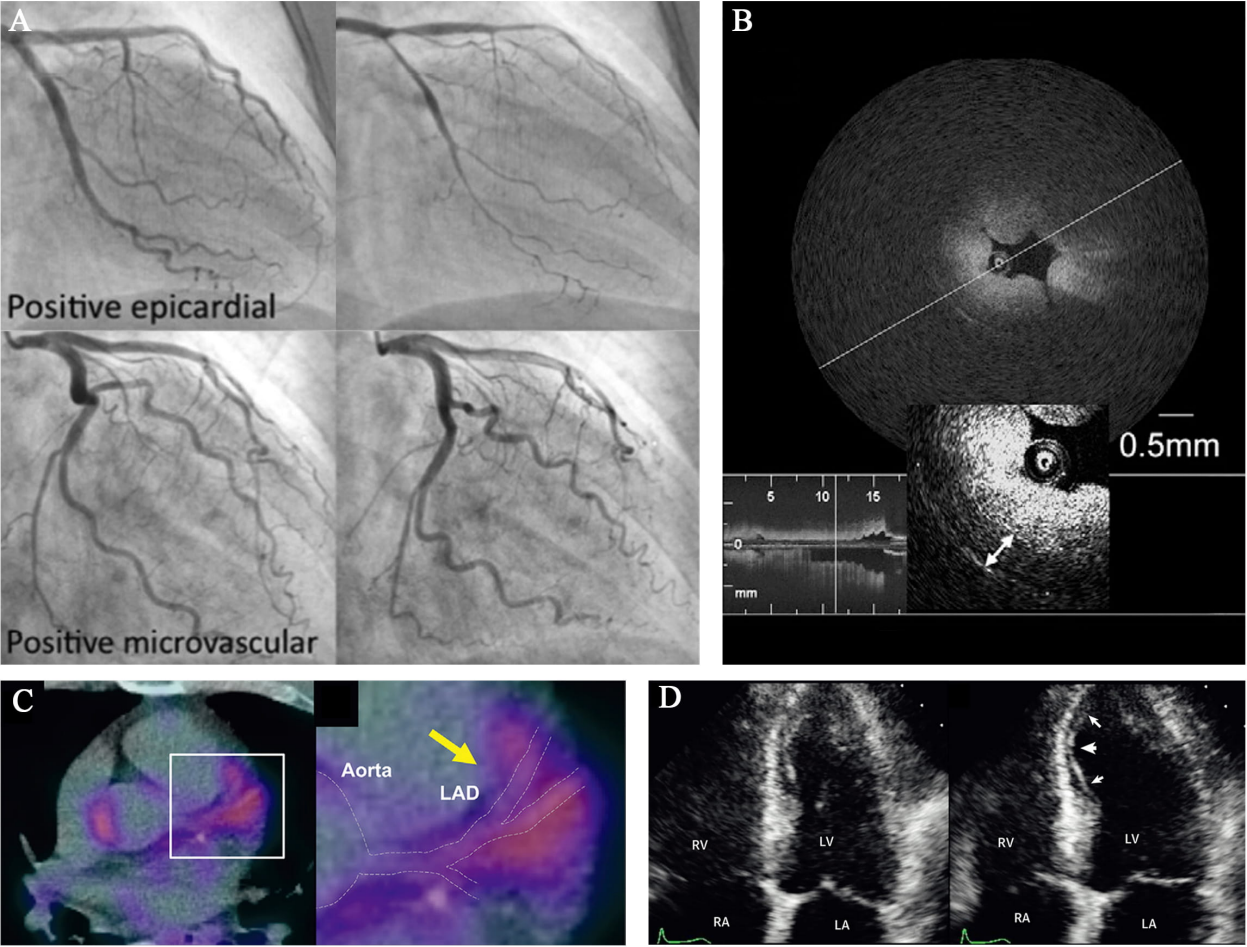
**Representative images of novel diagnostic approaches for CAS**. (A) Coronary angiograms of epicardial and microvascular CAS after spasm provocation test (SPT) using intracoronary perfusion of Ach. Images from Arrebola-Moreno *et al*. [131]. (B) Optical coherence tomography (OCT) image of a spasm lesion after provocation. Medial thickening led to luminal narrowing with intimal gathering. Image from Tanaka *et al*. [128]. (C) ^18^F-fluorodeoxyglucose (FDG) positron emission tomography/computed tomography (PET/CT) image of a CAS patients. FDG uptake of coronary PVAT was significantly increased. Image from Ohyama *et al*. [109]. (D) Myocardial contrast echocardiography (MCE) was carried out with intravenous injection of ergonovine. Apparent regional wall motion abnormalities (arrows) of the interventricular septum and left ventricular (LV) apex, compared with the resting state (left image). Images from Om *et al*. [135].

Since the spontaneous coronary vasospasm at the time of angiography is only occasionally observed [134], the current gold-standard diagnosis of CAS is documentation by angiography with pharmacological provocative testing *via* high-dose intracoronary administration of Ach, ergonovine, or methylergonovine [2]. The typical positive response should include a transient >90% vasoconstriction (Fig. 3A, Ref. [109, 128, 131, 135]) with reproduction of the usual chest pain and ischemic ECG changes at the meantime [2]. Abnormalities of ventricular wall motion on echocardiogram is considered to be equivocal for CAS as well [119]. To distinguish from obstructive arthrosclerosis and other underlying acute coronary syndrome [136], standard 12-lead ECG during an attack, ambulatory cardiac monitoring, or exercise stress testing should be initially performed in a standard cardiac workup [11]. Although coronary artery SPT has been clinically practiced for 40 years [2], complications by invasive operations like arrhythmias (6.8%) [137], hypertension, hypotension, and nausea [138] should also be noteworthy. Therefore, the procedure is suggested to be performed in a specialized center after careful evaluation of the risks and benefits [2], which limits the accessibility and restricts progress of CAS for decades.

#### 4.1.2 Coronary Angiography (CAG) 

CAG remains the gold standard for CAD [117]. However, except from the occasional attacks, the coronary artery shows normal appearance on resting CAG [116]. Therefore, if a patient is suspected with CAS, the angiography always accompanies with provocation testing to document the coronary spasm [134]. However, it is challenging to evaluate the interplay of the functional aspects and structural ones in patients with coronary artery atherosclerosis and the provocation testing is usually not performed in the presence of a significant epicardial stenosis. But studies approve that spontaneous attacks of coronary spasm can be superimposed on a relevant stenosis, illustrating the missing part in present clinical practice [118].

#### 4.1.3 Electrocardiogram (ECG)

An ECG of CAS diversifies from completely normal to ST deviation, T, U, R wave abnormality and arrythmia, depending on the severity, duration of episodes and distribution of the spasm artery [119, 120]. Mild seizures could appear just normal in ECG, while total or subtotal spasm of a major coronary artery tend to cause a ST-segment elevation in the leads [120]. However, ST-segment depression also occurs when a less severe, subendocardial myocardial ischemia occurs, when a major artery receiving collaterals or a small artery is completely occluded [122]. These situations include most part of unstable angina/non–ST-elevation myocardial infarction (NSTEMI) cases, thus making ST-segment depression more frequent in CAS [14]. A previous study has shown that 45% of patients with angina at rest and ST-segment depression alone had CAS [123].

In addition to ST-segment changes, a peaked and symmetrical T wave appears in around 50% of cases during a focal proximal coronary spasm [119]. And other wave changes can occur including a delay in the peak and an increase in the height and width of R wave, a decrease in magnitude of S wave and negative U wave may also appear [22]. Various forms of arrhythmia including ventricular premature complex, ventricular tachycardia and/or fibrillation (mostly in case of anterior ischaemia), atrioventricular block (mostly in case of inferior ischaemia), asystole and supraventricular tachyarrhythmias may also be present [121]. In conclusion, ECG takes its advantage in convenience, safety, availability and high-acceptability.

However, even with ambulatory ECG monitoring, the attack may not appear during the monitoring periods, especially when the attack is not frequent [139]. Moreover, ECG does not provide direct or specific evidence of CAS [22]. Thus ECG monitoring is an auxiliary detection in clinic.

#### 4.1.4 Intracoronary Imaging

Intracoronary imaging, such as optical coherence tomography (OCT) and intravascular ultrasound (IVUS) [117], is capable of addressing not only the morphological changes of intima and media during vasospasm, but also providing information regarding the association of vasospasm with underlying atherosclerotic plaque, fibrous cap disruption, enhanced adventitial vasa vasorum [125, 127, 140], increased PVAT volume [109], inflammation, erosion or thrombus formation [119]. OCT analysis during CAS reveals a typical image of intimal bumps deforming the lumen, combining with intimal gathering (Fig. 3B), without alteration of the intimal area. Medial contraction is presented by an increment in medial thickness [124]. However, intracoronary imaging does not wildly spread in clinical practice due to the complex procedure and low specificity, and each approach has its advantages and disadvantages. OCT has better image quality and resolution, which enables estimations of intima [125, 126]. IVUS has a deeper penetration (4–8 mm versus 2 mm of OCT), which assists accessing perivascular injury. In addition, it is safer and easier to perform IVUS since there is no need to cut off the blood flow, rather than OCT which still needs an interruption [126].

#### 4.1.5 Positron Emission Tomography (PET)

PET is a well-validated technique that can not only help assess coronary vasomotor function by providing non-invasive, accurate, and reproducible quantification of myocardial blood flow and coronary flow reserve (CFR) in humans, but also assist in revelation of coronary spasm tissue image [130]. Intriguingly, inflammatory changes of coronary PVAT assessed by ^18^F-FDG PET imaging (Fig. 3C) were more extensive at the spastic segments of CAS patients as compared to control subjects, which showed significantly suppression after CCBs treatment [109]. Hence, aside from the high price, PET/CT might be useful to assess coronary artery function and the perivascular tissue inflammation surrounding the coronary arteries.

#### 4.1.6 Myocardial Contrast Echocardiography (MCE)

This non-invasive technique is able to provide indirect functional information about micro vessels and thus assists in diagnosing CAS (Fig. 3D). Ong *et al*. [132] documented a transient myocardial ischemia by myocardial contrast echocardiography during Ach-induced CAS. Similarly, Arrebola-Moreno *et al*. [131] has shown the MCE as a systematic evidence for 60% Ach-induced CAS, consistent with single photon emission computed tomography (SPECT) and ECG. However, there are still many limitations in MCE. Due to the restriction of supine position that all the transthoracic echocardiographic images are performed at, it is possible for operators to ignore the minor systolic wall motion [131]. Furthermore, MCE can only detect tissue perfusion in the addition of extra contrast because of the poor back scattering from red blood cells [130], which impairs specificity of the technique. In fact, few available studies of MCE are focused on CAS since the vast majority pay attention to the vasodilatation dysfunction [133].

### 4.2 Serum Biomarkers

Recently, non-invasive biochemical markers have been found to associate with the occurrence of CAS [141], including inflammatory factors, Lipoprotein a, Cystatin C, 5-HT, and ET-1 etc. (Table 2, Ref. [18, 29, 30, 49, 50, 66, 96, 101, 141, 142, 143, 144, 145, 146, 147, 148, 149, 150, 151, 152, 153, 154, 155, 156, 157, 158, 159, 160, 161, 162, 163, 164, 165, 166, 167, 168, 169, 170, 171, 172, 173]).

#### 4.2.1 Endothelial Dysfunction Markers

As mentioned above, ED has been demonstrated an underlying mechanism of CAS [45]. Several potential biomarkers are under investigation through this pathogenesis. It has been proved that cystatin C is a reliable marker of kidney dysfunction [142], and renal failure could lead to inactivation of eNOS [145], which is supposed to be a basic pathogenesis in CAS. In fact, 2 clinical studies conducted in Japan and Korea respectively found a promising relationship between a high level of cystatin C and the prevalence of CAS [143, 144]. Nevertheless, there are still questions since renal dysfunction is also related to atherosclerosis and CAD [141], thus further investigations are still required to identify cystatin C as the unique biomarker for CAS. Additionally, xanthine oxidoreductase (XOR) is a rate-limiting enzyme of purine metabolism, catalyzing the oxidation of hypoxanthine to xanthine and of xanthine to uric acid (UA) [146]. It has been elucidated that increased serum UA produces extra ROS [148], resulting in ED [144]. Previous studies also have revealed that XOR-induced ROS can lead to arterial smooth cell proliferation and migration, up-regulate the renin-angio-tensin system to cause vasoconstriction [147]. A recent prediction model including XOR activity showed significantly improved C index (0.771 versus 0.685 of baseline model), net reclassification index (0.612; 95% confidence interval, 0.237–0.986; *p *= 0.001) and integrated discrimination index (0.098; 95% confidence interval, 0.040–0.156; *p *= 0.001), and concluded that serum XOR level might be an effective biomarker of CAS [29].

#### 4.2.2 Inflammatory Markers 

Within the belief of an association between inflammation [64], vasomotor dysfunction [45] and CAS, researchers keep finding evidence to prove inflammation markers as potential predictors for CAS, such as hs-CRP and soluble CD40 ligand (sCD40L). Hung *et al*. [149] showed that serum hs-CRP concentrations were correlated independently to CAS in 116 Taiwanese patients with VSA (41% with focal spasm) versus 66 control patients. Teragawa *et al*. [150] reported that increased serum hs-CRP levels were an independent predictor of coronary microvascular dysfunction by assessing coronary blood flow responses to Ach. Masami *et al*. [148] found hs-CRP were significantly increased in the VSA group (N = 441) than in the atypical chest pain group (N = 197). Ong *et al*. [18] found elevated hs-CRP and sCD40L concentrations were significantly (*p *≤ 0.05) associated in patients with angina pectoris free from angiographically obstructed coronary arteries. However, there is no obvious correlation between neopterin and CAS since it plays a role in the presence and progression of obstructive CAD [18]. Furthermore, the clinical results about inflammatory factors remain contradictory as a Korean study turned out to show that patients with CAS had no difference in levels of serum CRP as compared to those without CAS. Meanwhile the level of peripheral monocyte counts is found as a good potential marker for CAS [151].

#### 4.2.3 Vasoactive Markers

Except from hs-CRP and sCD40L as mentioned above, more biomarkers are found to be associated with CAS *via* inducing vasomotor dysfunction since decades ago. In 1990s, several laboratory teams viewed successively that the levels of ET-1 increased in blood during the episodes of CAS [30]. And bosentan, an antagonist of endothelin receptor, significantly relieved the severity and frequency of chest pain induced by CAS [152]. Until now, the relationship and pathogenesis of ET-1 in CAS almost disclose, but the clinical utility of ET-1 as a biomarker of the diseases is still on the way. In addition, 5-HT is proved to play an important role in vasocontraction and vasodilation [174]. Researchers found a high level of 5-HT in blood of patient with CAS during episodes as well as nonischemic intervals [153]. A recent study conducted showed an elevation of 5-HT in CAS patients without obstructed arteries [154]. Fortunately, no obvious contradictions occur in various studies so far. But there are still more work needing to be done about 5-HT before it gets to be applied in clinical practice because of lack of fresh evidence and clinical utility tests. Moreover, recent clinical studies found endogenous neuropeptide Y, another effective vasoactive factor, as a potential pathogenesis of CAS especially microvascular constrictions, for both patients without coronary stenosis and patients of ST-elevated myocardial infarction [155]. Intriguingly, as a co-transmitter of norepinephrine, neuropeptide Y is the only biomarker conformed to be correlated to microvascular spasm instead of epicardial ones [141], which indicates the potential differentiation between spasm in two sizes of coronary arteries and underlying different corresponding medication. Obviously, it will take a further more time from confirming the significant correlation between neuropeptide Y and CAS, to identify it as a well-qualified biomarker for clinical use.

#### 4.2.4 Abnormal Perivascular Adipose Tissue Metabolism

Tsuchida *et al*. [158] have already reported that higher lipoprotein(a) level was associated with coronary vasomotion in VSA. Masami *et al*. [148] verified the relationship between serum lipoprotein(a) level and VSA again within 441 Japanese patients. Intriguingly, it has been suggested that the lipoprotein(a) level is related to racial and genetic backgrounds [159], which suggest it is difficult to control the lipoprotein(a) level with medications for the management of VSA in some way. However, a large-scale clinical study did not identify obvious relationship between lipoprotein(a) and the vasospastic response to the intracoronary Ach provocation test [157].

#### 4.2.5 RhoK Activity in Circulating Neutrophils 

Accumulated evidence proves that enhanced RhoK activity plays a central role in the coronary VSMC hypersensitivity, which we have demonstrated in CAS pathogenesis above [50, 162]. Further investigations suggest that RhoK activity in circulating neutrophils maybe a potential biomarker for coronary spasm both in diagnosis and assessment of disease activity and efficacy of treatment [164]. In fact, a previous study showed an immediate, temporary increase of RhoK activity in circulating neutrophils in VSA patients after the Great East Japan Earthquake due to disaster-related mental stress [160]. And the cross-link between stress and CAS is indicated by another experimental study which found excessive sensitivity of VSMC to 5-HT under exposure to sustained elevation of serum cortisol level, resulting in coronary vasoconstrictive responses in pigs *in vivo * [66]. Moreover, there are some interesting biological coincidence between RhoK and CAS. For example, researchers found a circadian variation of RhoK activity in circulating neutrophils with a peak in the early morning, which showed strong association with alterations in coronary basal tone and vasomotor reactivity and might explain the onset preference of CAS [49]. Furthermore, the suppression effect on RhoK by estrogen may partly account for the higher incidence of vasospastic disorders in postmenopausal women [161]. Finally, RhoK activity in circulating neutrophils combining with the Japanese Coronary Spasm Association (JCSA) risk score substantially appears to be a better prognostic choice in risk stratification of VSA patient as compared with either alone [165]. Taking these issues into consideration, it seems that RhoK activity in circulating neutrophils has a strong potential to be developed into a useful biomarker for CAS with a broad versatility. Further investigations about mechanism, stability, detection time window and simplified measurement are required before it being applied to patients.

#### 4.2.6 Oxidative Stress 

Oxidation of low density lipoprotein (LDL) produces ox-LDL, which has been proven as a well-established marker of oxidative disorder [141]. Meanwhile, oxidation of LDL is also a key factor in the process and plays a role throughout atherosclerosis as well as CAS pathogenesis [167]. Recently, malondialdehyde-modified low-density lipoprotein (MDA-LDL) is suggested as another marker of endothelial damage [168]. Observational studies reported a strong correlation between serum MDA-LDL levels and endothelial damage, assessed with flow-mediated dilatation [168]. High MDA-LDL levels harbor a predisposing atherosclerotic segment for coronary spasm to arise, which explains the higher chances of ergonovine-induced CAS [166]. MDA-LDL lowering therapy such as intensive statin treatment [169] may have the potential to treat CAS.

#### 4.2.7 Circulating MicroRNAs

Human microRNAs (miRs) are small, single-stranded, endogenous noncoding RNAs that regulate gene expression at the post-transcriptional level by promoting the messenger RNA (mRNA) degradation or repressing certain coding mRNA translation [127]. It is recently reported that the significant higher expression levels of circulating miR-17-5p, miR-92a-3p, and miR-126-3p show discriminatory power in distinguishing patients with VSA from other CADs [170]. MiRs above are indicated to inhibit eNOS expression directly or *via KLF2* gene [170, 171], resulting in impaired NO production and thus leaving the coronary arteries in risk of vasoconstriction, platelet aggregation, low-density lipoprotein metabolic abnormalities and VSMC proliferation disorder [172, 173].

## 5. Conclusions

During the last decades, our knowledge of CAS has been increasingly progressed due to advances in the research strategy and diagnostic approaches. This review summarized the clinical risk factors and molecular mechanisms of CAS pathogenesis, and introduce state-of-the-art diagnostic strategies including both clinical imaging approaches and currently under laboratory-testing biomarkers. More mechanistic studies are mandated to further uncover the development of CAS. The seemingly promising biomarkers exist contradictory results, which suggests a long way off from reaching the clinical practice. More rigorous studies are required for further improvement.
